# The *Schizosaccharomyces pombe* Glycosyltransferase Gmh5 is a Functional Homologue of the α-1,6-Mannosyltransferase Mnn10 Crucial for N-Glycan Processing

**DOI:** 10.17113/ftb.64.01.26.9195

**Published:** 2026-02-15

**Authors:** Mark Lommel, Franziska Hutzler, Lina Siukstaite, Klemens Wild, Antonija Grbavac, Irmgard Sinning, Sabine Strahl

**Affiliations:** 1 Centre for Organismal Studies (COS), Heidelberg University, Im Neuenheimer Feld 360, 69120 Heidelberg, Germany; 2 Heidelberg University Biochemistry Center (BZH), Im Neuenheimer Feld 328, 69120 Heidelberg, Germany; # Present Address: Saarland University, Department of Microbiology, Campus A1 5, 66123 Saarbrücken, Germany; § Present Address: New York University, Department of Molecular Pathobiology, New York, USA

**Keywords:** glycosyltransferase Gmh5, mannosyltransferase, N-glycosylation, O-mannosylation, *Schizosaccharomyces pombe*, cell wall integrity

## Abstract

**Research background:**

Glycosyltransferases represent a large and diverse family of enzymes that catalyze the transfer of sugar residues to proteins and lipids, thereby regulating essential cellular processes such as protein quality control and cell wall biosynthesis. In yeast, protein O-mannosyltransferases and other glycosyltransferases are crucial for maintaining cell wall integrity. While the functions of many of these enzymes are well characterized, the role of some of them, such as Gmh5p, remains unknown. This study aims to elucidate the function of Gmh5p, a previously uncharacterized member of the GT34 glycosyltransferase family, in the context of protein and cell wall biosynthesis in *Schizosaccharomyces pombe*.

**Experimental approach:**

To identify proteins and pathways compensating for reduced O-mannosylation, we performed a genetic screening for multicopy suppressors in a conditional lethal nmt81-*oma2*^+^ mutant background. The enzymatic activity of Gmh5p was biochemically characterized, and its functional homology to known mannosyltransferases was assessed through complementation experiments in *Saccharomyces cerevisiae*. In addition, the N-glycosylation status of model substrates was analyzed in gmh5Δ mutant strains.

**Results and conclusions:**

Gmh5p was identified as a suppressor of O-mannosylation defects. Contrary to its predicted function, Gmh5p did not exhibit α-1,2-galactosyltransferase activity but instead showed mannosyltransferase activity. Expression of *gmh5*^+^ in *S. cerevisiae* mnn10Δ mutants restored hygromycin tolerance to near wild-type levels. Furthermore, N-glycosylation of model substrates was reduced in gmh5Δ mutants. These results demonstrate that Gmh5p is a mannosyltransferase involved in the outer chain elongation of N-linked glycans and functions as a homologue of Mnn10p.

**Novelty and scientific contribution:**

This study provides the first functional characterization of Gmh5p as a mannosyltransferase of the GT34 family and demonstrates its role in N-glycan biosynthesis. Our findings expand the current understanding of the diversity and specificity of glycosyltransferases in eukaryotes and highlight their importance in cell wall biology.

## INTRODUCTION

Glycosylation is among the most diverse and complex post-translational modifications of secretory and membrane proteins. The structural diversity of glycans not only modulates the physicochemical properties of proteins but also plays crucial roles in a wide range of cellular processes, including protein quality control, cell signalling, and host-microbe interactions (*1*).

In the eukaryotic model organism budding (*Saccharomyces cerevisiae*) and fission yeast (*Schizosaccharomyces pombe*), most proteins that enter the secretory pathway are subject to N-glycosylation and O-mannosylation (*2*, *3*). Both modifications are essential for cell growth and division, particularly due to their importance in building and maintaining an intact cell wall (*4*). N-glycosylation begins at the endoplasmic reticulum (ER) membrane, where the lipid-linked oligosaccharide (LLO) Glc_3_Man_9_GlcNAc_2_-PP-dolichol is assembled in a stepwise manner (*5*). The completed oligosaccharide is then transferred en bloc from the LLO to asparagine residues within the consensus sequence Asn-X-Ser/Thr of nascent polypeptides, a reaction catalyzed by the oligosaccharyltransferase complex. During ER protein quality control, specific glycosidases remove the three glucose residues and, optionally, one mannose residue from the protein-linked oligosaccharide (*5*). Correctly folded glycoproteins can then exit the ER and enter the Golgi apparatus, where N-linked glycans are further processed. While the ER-based steps of N-glycan biosynthesis are highly conserved among eukaryotes, subsequent modifications in the Golgi apparatus are diverse and species-specific (*5*). In all studied yeasts, the core N-glycan is extended by a single α-1,6-mannose residue, added by the α-1,6-mannosyltransferase Och1p (*6*, *7*). In *S. cerevisiae*, this mannose can be further elongated by α-1,6-linked mannose units, catalyzed by the M-Pol I complex, primarily composed of Mnn9p and Van1p (*8*). Additional mannosyltransferases of the M-Pol II complex (including Anp1p, Mnn10p, Mnn11p and Hoc1p in *S. cerevisiae*) assemble a polymannose backbone, which can reach up to 50–150 mannose residues in baker’s yeast, though the exact length can vary (*9*, *10*). This α-1,6-polymannose backbone is further decorated by α-1,2- and α-1,3-mannosyltransferases, resulting in the addition of short side chains that contribute to the structural diversity of yeast N-glycans (*10*). In contrast to baker’s yeast, in *S. pombe*, complex N-linked galactomannans with structures such as Hex_10-15_GlcNAc_2_ are formed (*11*). An M-Pol II-like complex composed of Mnn9p and Anp1p, sequentially elongates the mannose chain initiated by Och1p (*7*, *12*). However, in contrast to *S. cerevisiae*, the outer chain of N-glycans in *S. pombe* is mainly decorated with single α-1,2-galactose residues, which can be further modified by pyruvylated β-1,3-linked galactose (*13*). Several α-1,2- and α-1,3-linked galactose units may also be incorporated (*14*, *15*). The α-1,2-galactosyltransferases Gma12p, Gmh2p, Gmh3p and Gmh6p are involved in these modifications, with evidence suggesting that they have different substrate preferences (*15*). Additional glycosyltransferases, such as Otg2p and Otg3p, can form α-1,3-linkages between galactose and mannose residues, contributing further to glycan diversity (*16*). Furthermore, recent studies confirm that Otg1p exhibits α1,3-galactosyltransferase activity specifically when co-expressed with Gmh6p, demonstrating that its enzymatic function requires the presence of α-1,2-linked galactose provided by this interaction (*17*).

O-mannosylation is initiated in the ER lumen by members of the protein O-mannosyltransferase (PMT) family, which is conserved from fungi to humans (*3*, *4*). These enzymes transfer mannose from dolichol phosphate-mannose to the hydroxyl group of serine or threonine residues on nascent polypeptides. The PMT family is divided into three subfamilies: PMT1, PMT2 and PMT4 (*4*). In *S. pombe*, each subfamily is represented by a single member (Oma1p, Oma2p and Oma4p) (*18*). Functional conservation between fission and budding yeast has been demonstrated, and heteromeric complexes (Oma1p/Oma2p) differ in substrate specificity from Oma4p (*18*). After the initial addition of mannose, properly folded glycoproteins exit the ER. In the Golgi, O-mannosyl glycans are further extended in a species-specific manner. In *S. cerevisiae*, the O-linked mannose can be elongated by α-1,2- and α-1,3-mannosyltransferases from the KTR and MNN1 families (*19*). In *S. pombe*, O-mannosyl glycans are typically elongated with one or two α-1,2-linked mannose units (*14*) and may be further decorated with α-1,2- and α-1,3-linked galactose residues, catalyzed by α-1,2-galactosyltransferases (Gma12p, Gmh2p, Gmh6p) and the α-1,3-galactosyltransferase Ogh2p (*15*, *16*).

In sum, the complex glycosylation pathways in yeasts result in large, structurally diverse N-linked glycans and short, highly abundant O-mannosyl glycans. These modifications are critical for protein function and for the biogenesis and integrity of the yeast cell wall, which is a vital structure (*20*). In *S. pombe*, the cell wall consists of an inner layer of α- and β-glucan covered with galactomannoproteins (*21*). Mutants with altered galactomannan content display phenotypes similar to those with defects in O-mannosylation. For example, deletion of *oma2*^+^ in *S. pombe* is lethal, while oma1Δ and oma4Δ mutants are viable but exhibit growth defects at elevated temperatures and in the presence of cell wall stressors such as caffeine (*18*). These mutants also show severe abnormalities in cell wall and septum formation, underlining the essential role of O-mannosylation in cell wall integrity.

To learn more about the molecular causes underlying the phenotypes of omaΔ mutants, we screened for multicopy suppressors of the conditional lethal nmt81-*oma2*^+^ mutant in this study. We identified the yet functionally uncharacterized predicted α-1,2 galactosyltransferase Gmh5p. Strikingly, we provide evidence that Gmh5p represents an orthologue of *S. cerevisiae* mannosyltransferase Mnn10p, an M-Pol II complex component.

## MATERIALS AND METHODS

Unless stated otherwise, all medium components were obtained from Becton, Dickinson and Company (BD, Franklin Lakes, NJ, USA), and standard laboratory chemicals were purchased from either Merck KGaA (Darmstadt, Germany) or Carl Roth & Co KG (Karlsruhe, Germany).

### Yeast strains and growth conditions

The *Schizosaccharomyces pombe* and *Saccharomyces cerevisiae* strains used are listed in Table S1 (*14*, *18*). Rich media YES (yeast extract plus supplements) and YPD (yeast extract, Bacto™ peptone, dextrose) as well as minimal defined media EMM (Edinburgh minimal medium) and SD (synthetic defined) medium were used as described previously (*18*). Phosphate-free minimal medium based on EMM contained 14.6 mM sodium acetate instead of disodium hydrogen phosphate and potassium hydrogen phthalate. For shutdown experiments using the nmt81 promoter, cells were restreaked twice on EMM plates containing 5 mg/L thiamine. Standard procedures were used for yeast transformation as well as for genetic DNA manipulations. All cloning procedures were carried out in the *Escherichia coli* host DH5α (Thermo Fischer Scientific, Rockford, IL, USA).

*S. pombe* strains CMY1 and CMY2: a disruption cassette replacing the entire open reading frame of SPAC637.06 by the *kanMX* gene was amplified from genomic DNA of the heterozygous SPAC637.06 mutant strain BG_1883 (Bioneer Cooperation, Daejeon, South Korea) using primers Oligo 1305 and Oligo1306. A mass of 5 µg of the resulting DNA fragment was used to transform the wild-type (WT) yeast strain FY527 or the oma1Δ mutant strain SBY90. Transformants that integrated the disruption cassette were selected on YES medium containing G418. Genomic DNA was prepared from the resulting transformants and successful targeting of SPAC637.06 was confirmed by PCR using primers Oligo1231 and Oligo1232. All oligonucleotide sequences used in this study are available upon request.

### Multicopy suppressor screening and plasmids

The thiamine repressible nmt81-*oma2*^+^ strain TWY16 was transformed with *S. pombe* genomic libraries contained in pURSP1 and pURSP2, respectively (*22*). Before transformation, cells were grown in medium lacking thiamine. After transformation, cells were plated on selective medium lacking uracil and containing thiamine, then incubated for three days at 32 °C. For plasmid isolation, individual clones were grown overnight in 3 mL of cultures and harvested. Pellets were resuspended in 100 µL of buffer containing 50 mM Tris-HCl, pH=8.0, 50 mM EDTA, 8 % sucrose and 5 % Triton X-100. Cells were disrupted with glass beads (*d*=0.5 mm) using a RiboLyser (Hybaid, Altrincham, UK). After separation from the glass beads, samples were incubated for 3 min in a boiling water bath, chilled on ice and cell debris was pelleted for 10 min at 20 000×*g*. From the supernatant, plasmid DNA was recovered by ethanol precipitation and retransformed in *E. coli*. Isolated plasmids were retransformed in TWY16 and three independent clones were tested to reconfirm their suppressor ability on restrictive plates containing thiamine. To identify the genes contained in the suppressor plasmid, genomic sequences were determined by Sanger sequencing using M13fwd and M13rev universal primers.

Plasmids pTW51-1 and pTW60 have been described previously (*18*).

Plasmid pI-1/9: Plasmid pI-1/9 was directly derived from the multicopy suppressor screen with a genomic DNA fragment and only the *phx1*^+^ (SPAC32A11.03c) coding sequence.

Plasmid pCM6: The plasmid pII-1/2 containing the entire open reading frame of *mpg1*^+^ (SPCC1906.01) and a truncated coding sequence of SPCC1906.02c was isolated from the multicopy suppressor screen. This plasmid was cut with PstI to remove the SPCC1906.02c-containing fragment and religated, resulting in plasmid pCM6.

Plasmid pII-2/17: The plasmid pII-2/17 was directly derived from the multicopy suppressor screen and contains a genomic DNA fragment with only the *gmh5*^+^ (SPAC637.06) coding sequence.

Plasmid pCM10 and pCM11: For overexpression of *gmh5*^+^ and a *gmh5*^+^-hemagglutinin (HA) epitope-tag fusion in *S. pombe* and *S. cerevisiae,* primer pairs Oligo1246 combined with Oligo1247 or Oligo1248 were used to amplify a 1.0 kb DNA fragment containing the *gmh5*^+^ coding sequence. The resulting PCR products were cut with NotI and SalI or NotI and XhoI and subcloned into pREP3adh (*18*) cut with the same enzymes. The resulting plasmids pCM10 and pCM11 encode for Gmh5p and a Gmh5p-HA-tag fusion protein under the transcriptional control of the alcohol dehydrogenase 1 (*adh1*) promoter.

Plasmid pCM12: The 1.5 kb coding region of the *S. cerevisiae SUC2* gene was amplified by PCR from *S. cerevisiae* genomic DNA using Oligo1309 and Oligo1310. The primer pair was used to introduce a 5’ NotI restriction site and a 6× His epitope-tag and XhoI restriction site. The resulting PCR product was digested with NotI and XhoI and subcloned into pREP3adh cut with the same enzymes. The resulting plasmid encodes a Suc2-6xHis-3xHA fusion protein under the transcription control of the *adh1* promoter.

Plasmid pGma12HA: The primer pair Oligo1297 and Oligo1299 was used to amplify a 1.1 kb PCR fragment containing the coding region of *gma12*^+^ excluding the stop-codon. *S. pombe* genomic DNA was used as a template. The resulting PCR product was subcloned into pREP3adh using NotI and XhoI restriction sites. The resulting plasmid encodes for a Gma12p-3xHA fusion protein driven by the *adh1* promoter.

### Inhibitor sensitivity assays

Assays were performed in 96-well microtiter plates in a final volume of 200 µL of YES medium or YPD medium for *S. pombe* or *S. cerevisiae*, respectively. Serial dilutions of caffeine (0 to 17 mM; Sigma-Aldrich, Merck, St. Louis, MO, USA), hygromycin (0 to 16.25 µg/mL; Sigma-Aldrich, Merck) and the PMT-inhibitor R3A-5a (0 to 32 µM; Merck KGaA, Darmstadt, Germany (*23*)) were prepared in EMM or SD medium and inoculated from a logarithmically growing culture to a final absorbance *A*_600 nm_=0.02. The 96-well microtiter plates were incubated for 24 h at 30 °C and cell growth was determined by measuring the absorbance in a FLUOstar Omega (BMG Labtech, Ortenberg, Germany) microplate reader at 600 nm. All conditions were tested in triplicates and experiments were repeated twice.

### Preparation of membranes and cell walls

Crude yeast membranes used in Western blot analysis and mannosyltransferase assays were prepared as described previously (*24*). In brief, a total of cells corresponding to a combined *A*_600 nm_ of 200 were harvested from a logarithmically growing culture and washed once with ice-cold buffer containing 50 mM Tris-HCl, pH=7.4, and 5 mM MgCl_2_. Cells were resuspended in a buffer containing 50 mM Tris-HCl, pH=7.4, 5 mM MgCl_2_, and 1 mM phenylmethylsulfonylfluoride (PMSF), 1 mM benzamidine, 50 µg/mL tosyl-l-phenylalanine chloromethyl ketone (TPCK), 0.25 mM tosyl-l-lysine chloromethyl ketone (TLCK), 10 µg/mL antipain, 1 µg/mL leupeptin and 1 µg/mL pepstatin as protease inhibitors (PI). An equal volume of glass beads (*d*=0.5 mm) was added, and cells lysed in a RiboLyser (Hybaid; 4×25 s at level 4.5 with 1-minute intervals on ice). The tube was punctured at the bottom, and the lysate was allowed to flow into a fresh tube. Cell debris was removed by centrifugation (model 5430R; Eppendorf, Hamburg, Germany; 5 min at 1500×*g* and 4 °C). Microsomal membranes were collected by centrifugation (Eppendorf 5430R; 60 min at 30 000×*g* and 4 °C). The membrane fraction was resuspended in a buffer containing 20 mM Tris-HCl, pH=8.0, 10 mM EDTA, and PI equivalent to *A*_600 nm_ (cell)=1.0 per 1 µL. For cell wall preparations, cell lysates were pelleted for 20 s at 20 000×*g* and 4 °C, pellets were washed twice in ice-cold buffer containing 50 mM Tris-HCl, pH=7.4, and 5 mM MgCl_2_ plus PI and resuspended in the same buffer to 1.0 *A*_600 nm_ cell equivalents.

### Galactosyltransferase assay

Cultures of *S. cerevisiae* BY4741 strain transformed with pREP3adh (empty vector), pGma12 (*gma12*^+^) or pCM10 (*gmh5*^+^) were grown overnight in SD medium lacking leucine to final *A*_600 nm_ of 1.0. A total of cells corresponding to a combined *A*_600 nm_ of 200 was harvested by centrifugation (Eppendorf 5430R) at 3000×*g* for 10 min at 4 °C and washed once with TMS buffer (20 mM Tris-HCl, pH=7.5, 5 mM MgCl_2_ and 250 mM sucrose). Cell pellets were resuspended in 200 µL TMS buffer plus PI, and an equal volume of glass beads (*d*=0.5 mm) was added. Cells were lysed using a RiboLyser (Hybaid) in four 30-second intervals (level 4.5) interrupted by 1 min cooling on ice. The bottoms of the reaction tubes were punctured and lysates were collected. Cell debris was removed by centrifugation for 10 min at 3000×*g* and 4 °C and microsomal membranes were harvested from the supernatant at 30 000×*g* for 60 min at 4 °C. The membrane fraction was resuspended in TMS to a final concentration of 20 µg/µL, Triton X-100 was added to a final amount of 2 % (*m*/*V*) and microsomes were solubilized for 30 min at 4 °C with mild agitation. Solubilized samples were cleared by centrifugation at 30 000×*g* for 60 min at 4 °C, and the protein concentration was determined by DC protein assay (BioRad, Hercules, CA, USA). Then, galactosyltransferase assays of 50 µL of total volume were performed containing 0.1 M Hepes-NaOH, pH=7.0, 1 mM MnCl_2_, 25 nmol UDP-[3H]galactose (specific activity 1 mCi/mmol (1 Ci=3.7·10^10^ Bq); BioTrend, Köln, Germany), 5 µmol of a given sugar acceptor and 50 µg of solubilized protein extracts. Acceptors used were: α-methyl-d-mannoside (Sigma-Aldrich, Merck), α-methyl-d-galactoside (Sigma-Aldrich, Merck) and α-1,6-mannobiose (Sigma-Aldrich, Merck). The samples were incubated for 60 min at 30 °C. Each reaction was terminated by the addition of 200 µL of ice-cold water and loaded on an 800 µL Dowex-1 (Cl-form; Sigma-Aldrich, Merck) column packed in a Pasteur pipette. Each column was washed twice with 1 mL of water, eluates were combined, and radioactivity in the eluates was determined in a liquid scintillation counter (Beckman, Brea, CA, USA). Each reaction was performed in triplicate and the experiment was repeated twice.

### *Mannosyltransferase* a*ssay*

Cultures of *S. cerevisiae* BY4741 strain transformed with pREP3adh (empty vector) and pCM11 (Gmh5p*-*HA) were grown overnight in SD medium lacking leucine to final *A*_600 nm_=1.0. Microsomal membrane fractions were obtained as described above. Microsomes were resuspended in 300 µL buffer containing 20 mM Tris-HCl, pH=7.5, 5 mM MnCl_2_, 250 mM sucrose, 1 % digitonine, and PI, and solubilized for 1 h at 4 °C with mild agitation. Cell debris was removed by centrifugation (Eppendorf 5430R) at 20 000×*g* for 30 min at 4 °C and the supernatant was added to 50 µL of anti-HA monoclonal antibody covalently coupled to Sepharose (Roche, Mannheim, Germany). Proteins were immunoprecipitated overnight at 4 °C and washed three times with a buffer containing 50 mM HEPES, pH=7.2, 0.4 % digitonin and 10 mM MnCl_2_, and resuspended in 50 µL of the same buffer. Mannosyltransferase assays of 50 µL volume contained 50 mM HEPES, pH=7.2, 0.4 % digitonin, 10 mM MnCl_2_, 25 nmol GDP-[3H]mannose (specific activity 1 mCi/mmol (1 Ci=3.7·10^10^ Bq); BioTrend), 10 mM α-methyl-d-mannoside, and 5 µL of anti-HA sepharose. Reactions were incubated for 1 h at 30 °C with agitation. Reactions were terminated by adding 200 µL of ice-cold water and loaded on an 800 µL Dowex-1 (Cl-form, Sigma-Aldrich, Merck) column packed in a Pasteur pipette. Each column was washed twice with 1 mL of water, eluates were combined, and radioactivity in the eluates was determined in a liquid scintillation counter (Beckman). Each reaction was performed in triplicate and the experiment was repeated twice.

### Isolation and analysis of chitinase

For heterologous expression of the *S. cerevisiae* protein chitinase, *S. pombe* strains FY527, omh1Δ and CMY1 (gmh5Δ) were transformed with the plasmid pFML1-CTS1 (*14*), and chitinase was isolated as described elsewhere (*14*). In brief, 5 mL cultures were grown to saturation in EMM lacking leucine, harvested by centrifugation (Eppendorf 5430R) at 3000×*g* for 10 min at room temperature and resuspended in 10 mL EMM containing 0.1 % glucose and 2 % raffinose instead of 2 % glucose. Cultures were allowed to express chitinase for 48 h. The medium was then cleared by centrifugation (Eppendorf 5430R; 3000×*g* for 10 min at room temperature), 20 mg of crab shell chitin was added and the samples were incubated overnight at 4 °C with agitation. The chitin was pelleted by centrifugation (15 000×*g* for 15 min at 4 °C), washed three times with ice-cold buffer containing 50 mM Tris-HCl, pH=7.5, 150 mM NaCl, and chitinase was eluted in 50 µL 2× SDS sample buffer at 95 °C for 5 min. A volume of 10 µL of each sample was separated on 6 % SDS-PAGE and analyzed by Western blot.

### Isolation and deglycosylation of invertase

Strains FY127 (WT) and CMY1 (gmh5Δ) were transformed with plasmid pCM12 to heterologously express *S. cerevisiae* invertase Suc2p. Cell walls from these transformants were isolated as described above. To remove N-glycosidically linked glycans, cell walls of 5.0 *A*_600 nm_ equivalents were treated with endoglycosidase H (Endo H; New England Biolabs GmbH, Frankfurt am Main, Germany) according to the manufacturer’s instructions. Reactions omitting the glycosidase served as a control. From each reaction, proteins were extracted with 1× SDS sample buffer, and 1.0 *A*_600 nm_ cell equivalents were separated on SDS gels and analyzed by Western blot.

### Western blot analysis

Protein extracts were separated by SDS-PAGE and transferred to nitrocellulose membrane. Monoclonal mouse anti‐HA (16B12; Abcam, Cambridge, UK), polyclonal rabbit anti-Cts1 (gift from W. Tanner) and anti‐invertase (gift from L. Lehle) antibodies were used at 1:8000 (anti-HA) and 1:5000 (others) dilutions, respectively. A horseradish peroxidase conjugated secondary anti-mouse or anti-rabbit antibodies (Sigma-Aldrich, Merck) were used at 1:5000 dilution. Protein–antibody complexes were visualized by enhanced chemiluminescence using the Amersham ECL system (GE Healthcare, Chicago, IL, USA).

### Native gel electrophoresis of acidic phosphatase

Cells were grown in 5 mL YES medium to an *A*_600 nm_=1.5, and *N*(cell)=1.5·10^8^ were pelleted, resuspended in 5 mL of MM-P medium, and incubated for 3 h at 30 °C to induce production of acid phosphatase. The cells were then collected by centrifugation (Eppendorf 5430R; 3000×*g* for 10 min at 4 °C), washed once with 62.5 mM Tris-HCl, pH=6.8, and suspended in 200 μL ice-cold lysis buffer (62.5 mM Tris-HCl, pH=6.8, 1 mM EDTA, 2 mM PMSF, 0.1 mM dithiothreitol and 10 % glycerol). Cell lysates were prepared by vigorously mixing the cell suspension five times with glass beads (*d*=0.5 mm) for 30 s at 4 °C using a RiboLyser (Hybaid). The lysates were centrifuged at 15 000×*g* for 10 min, and the supernatants were recovered and centrifuged again at 15 000×*g* for 20 min. The supernatants were recovered again and protein was determined using a BCA™ protein assay kit (Thermo Fisher Scientific, Rockford, IL, USA). A mass of 30 μg of total protein from each sample were mixed with 1/3 volume of 0.01 % bromophenol blue, 15 % glycerol and 62.5 mM Tris-HCl, pH=6.8, and were immediately subjected to electrophoresis on a 6 % polyacrylamide native gel. The electrophoresis buffer and the method for staining for acid phosphatase activity were used as described elsewhere (*25*).

### Microscopy of S. pombe cells

Cells were grown overnight to an *A*_600 nm_ of 1.0. A volume of 1 mL of the cell suspension was harvested by centrifugation (Eppendorf 5430R) at 3000×*g* for 3 min. Cell pellets were washed once with distilled water and resuspended in 100 µL of staining solution containing either 0.5 mg/mL aniline blue (Sigma-Aldrich, Merck; dissolved in distilled water) or 5 µg/mL filipin complex (Sigma-Aldrich, Merck; dissolved in DMSO). Aniline blue served both as a β-1,3-glucan stain and as a vital dye for viability assays. Samples (15 µL) were mounted and analyzed on a Leica DM IBR fluorescence microscope (Leica, Wetzlar, Germany) equipped with Leica DFC 350 FX monochrome CMOS camera.

### Phyogenetic and structural analyses

Phylogenetic analysis of galactosyl- and mannosyltransferases was carried out using Clustal Omega (*26*). Phylogenetic tree data were visualized using Phylodendron (*27*). Input protein sequences used were: *S. cerevisiae*: ScHoc1 (UniPro P47124), ScMnn10 (UniProt P50108), ScMnn11 (UniProt P46985), ScMnn9 (UniProt P39107), ScAnp1 (UniProt P32629); *S. pombe*: SpGmh1 (PomBase SPAC5H10.1), SpGmh2 (PomBase SPAC5H10.13c), SpGmh3 (PomBase SPAC22E12.06c), SpGmh4 (Pombase SPBC8D2.17), SpGmh5 (PomBase SPAC637.06), SpGmh6 (PomBase SPBC1289.13c), SpGma12 (PomBase SPCC736.04c); *Candida albicans*: CaMnn10 (UniProt Q59UR2); *Kluyveromyces lactis*: KlMnn10 (UniProt Q70KX5).

Superimpositions were performed with program Coot (*28*) and structure predictions were done with AlphaFold (*29*). Structural models and their superimpositions were visualized using PyMOL (*30*).

### Statistical analyses

Statistical analyses were performed using GraphPad Prism v. 8.0 (*31*). For glycosyltransferase assays data were evaluated using one-way ANOVA followed by Tukey´s *post-hoc* test to create confidence intervals for all pairwise differences between the different conditions. For inhibitor assays, non-linear expression analysis was performed and IC_50_ values were determined. Differences in IC_50_ values across different conditions were determined by one-way ANOVA followed by a Tukey´s *post-hoc* test.

## RESULTS AND DISCUSSION

### Identification of gmh5^+^ as multicopy suppressor of decreased O-mannosylation

**Fig. 1:**
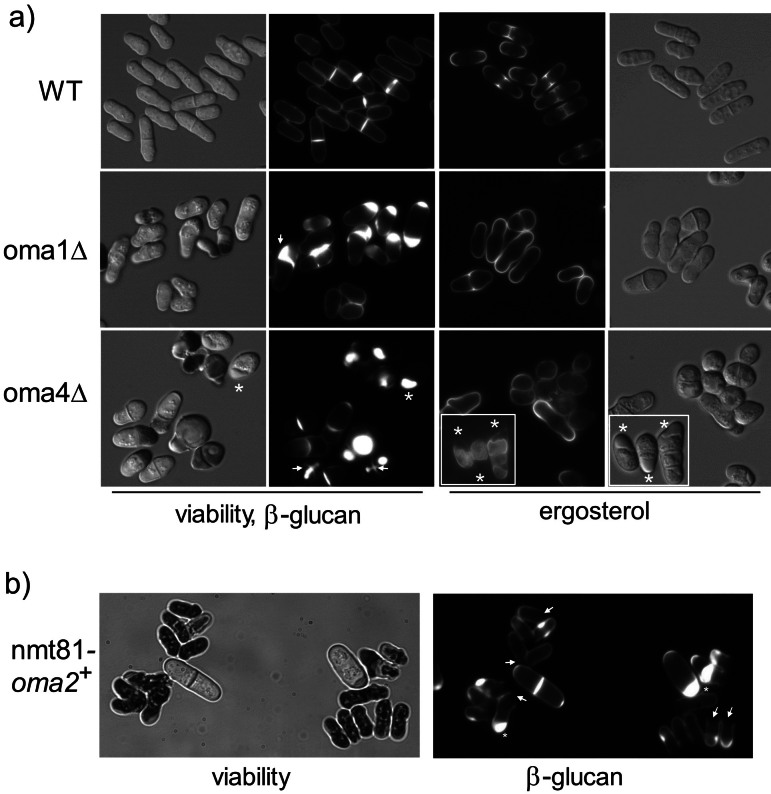
Phenotypic characterization of *Schizosaccharomyces pombe* oma mutants: a) aniline blue (cell wall β-1,3-glucan and dead cells) and filipin (ergosterol, sterol-rich regions of the plasma membrane) staining of wild-type (WT) and mutants oma1Δ and oma4Δ, grown to logarithmic phase on EMM medium (for details see Materials and Methods). Mutant cells show abnormal thickening of the cell wall by β-1,3 glucan depositions at the poles (asterisk) or the septum (arrows), and b) aniline blue staining of nmt81-*oma2*^+^ cells grown on thiamine-containing medium. Twenty-four hours after shut-off of the promoter most cells are dead (dark cells, left panel) and/or show β-1,3 glucan depositions in the cell wall (right panel). Differential interference contrast (DIC) and fluorescence microscopic images are shown at 630-fold magnification

**Fig. S1:**
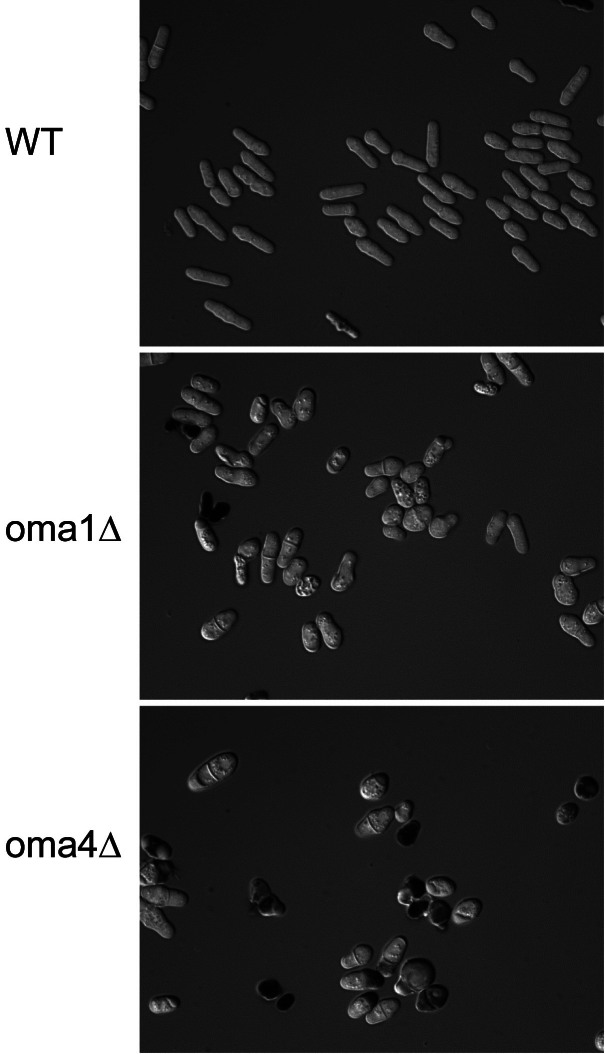
Viability of *Schizosaccharomyces pombe* omaΔ mutants. Wild-type (WT), oma1Δ and oma4Δ mutants grown to logarithmic phase on EMM medium are shown. Dead cells are visualized by aniline blue staining. Differential interference contrast (DIC) images are shown; magnification 630-fold

We previously demonstrated that in the model yeast *S. pombe* decreased O-mannosylation severely affects cell wall morphology and stability, especially during septum formation (*18*). To characterize these defects in more detail, logarithmically growing WT, oma1Δ and oma4Δ mutant cells were stained with aniline blue. The dye preferentially binds to linear β-1,3-glucan (*32*), mainly localized in the primary septum of dividing cells, where it can be detected from initiation to completion of septum formation (*33*). In contrast to WT cells with defined staining of the septum, around 30 % of oma1Δ (out of 847 cells) and 40 % oma4Δ (out of 261 cells) cells, respectively, showed significant accumulation of β-1,3-glucan at one or both cell poles, or at the septum (Fig. 1a). Staining of sterol-rich areas of the plasma membrane confirmed local thickening of the cell wall in these mutants (Fig. 1a). Furthermore, vital staining with aniline blue revealed that around 7 % (30 out of 455 cells) of oma1Δ and 20 % (31 out of 153 cells) of oma4Δ cells die during vegetative growth (Fig. S1).

To define the lethal phenotype caused by defective O-mannosylation more precisely, we took advantage of *oma2*^+^, the essential member of the PMT family in *S. pombe* (*18*). We analyzed a conditional lethal mutant expressing the *oma2*^+^ gene under the control of the nmt81 promoter, which is repressed by thiamine (*34*, *35*). As shown in Fig. 1b, after 24-hour incubation in thiamine-containing medium, more than 80 % of the nmt81-*oma2*^+^ cells were dead (210 out of 253 cells) with most cells lysing at the septum during or after cell separation (170 out of 253 cells). In some cells, large β-1,3-glucan depositions could be observed in the cell wall, similar to oma1Δ and oma4Δ cells.

In *S. pombe*, β-1,3-glucan serves as a primary structural component of the cell wall (*21*). When the synthesis of other cell wall constituents, such as α-glucan or glycoproteins, is compromised, the cell activates compensatory mechanisms to maintain integrity (*21*). Central to this response is the upregulation of β-1,3-glucan synthesis, which is regulated by signalling pathways involving Rho1 GTPase and protein kinase C homologues, which respond to cell wall stress (*36*). This adaptive increase in β-glucan content reinforces the cell wall, ensuring cellular morphology and viability under stress conditions. The accumulation of β-glucan at the septum and cell poles and frequent cell lysis at the septum in O-mannosylation mutants (Fig. 1a and Fig. 1b) highlights the crucial role of O-mannosylation in cell wall organization. Consistent with this interpretation, transcriptome analyses of mutant oma421, expressing the *oma1*^+^, *oma2*^+^ and *oma4*^+^ genes under the control of the nmt81 promoter, indicated an induction of genes related to Rho1/PKC signalling pathways when grown in the presence of thiamine. A detailed description of these data will be presented elsewhere.

**Fig. 2:**
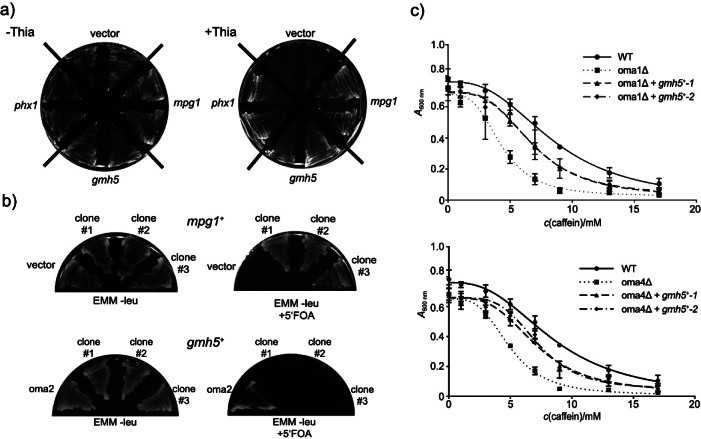
Multicopy suppressors of O-mannosylation deficient mutants: a) complementation of synthetic lethality of mutant nmt81-*oma2*^+^. Mutant strain nmt81-*oma2*^+^ (TWY16) transformed with the plasmid pI-1/9 (*phx1*^+^), pCM6 (*mpg1*^+^), pII-2/17 (*gmh5*^+^) or pUR19 (vector), was grown on EMM plates without uracil in the absence (-Thia, left panel) or presence (+Thia, right panel) of thiamine. Shown are plates with two individual transformants of each plasmid grown at 32 °C for three days. In contrast to the vector control, *phx1*^+^, *mpg1*^+^ as well as *gmh5*^+^ support the vegetative growth of the cells after nmt81-*oma2*^+^ was switched off under the applied conditions (for details see Materials and Methods). The slight growth of the vector control may be due to residual expression of *oma2*^+^ resulting from promoter leakiness under thiamine repression, b) complementation of the lethality of mutant oma2Δ. Strain TWY14 (oma2Δ expressing *oma2*^+^ on the ura4^+^ plasmid pTW51-1) was transformed with plasmid pTW60 (LEU2, *oma2*^+^), pREP3adh (LEU2, vector), pCM10 (LEU2, *gmh5*^+^) and pMLP02 (LEU2, *mpg1*^+^). For plasmid loss experiments, transformants were grown on EMM plates without leucine, in the absence (left panel) or presence (right panel) of 5-FOA. Shown are plates with three individual transformants of the genes of interest grown at 32 °C for three days. Cells expressing *oma2*^+^ on plasmid pTW60 (lower panel) or the suppressor *mpg1*^+^ on pMLP02 (upper panel) are able to lose plasmid pTW51-1 (*ura4*^+^, *oma2*^+^), thus grow in the presence of 5-FOA. In contrast, expression of the suppressor *gmh5*^+^ (pCM10) does not compensate for the complete loss of Oma2p activity. Thus, like the vector control (upper panel), *gmh5*^+^ expressing cells are unable to lose plasmid pTW51-1, and therefore do not grow on 5-FOA-containing plates (lower panel), and c) overexpression of *gmh5*^+^ decreases caffeine sensitivity of oma1Δ (upper panel) and oma4Δ (lower panel) mutants. Viable oma1Δ and oma4Δ mutants were transformed with the vector control pREP3adh (omaΔ), pCM10 (*gmh5*^+^) or the corresponding oma gene (WT; pTW24 (*oma1*^+^) or pTW25 (*oma4*^+^)); omaΔ + *gmh5*^+^-1 and omaΔ + *gmh5*^+^-2 represent two independent transformants. Growth in the presence of increasing amounts of caffeine as indicated was tested as detailed in Materials and Methods. Each experiment was performed twice using three technical replicates. Results were used for nonlinear regression analysis and IC_50_ was determined. Differences in the IC_50_ values were confirmed to be significant by statistical analysis (p<0.05). WT=wild-type

To find out which proteins and pathways are affected when O-mannosylation is decreased, we screened for multicopy suppressors of the conditionally lethal phenotype of mutant nmt81-*oma2*^+^. The *S. pombe* genomic DNA libraries pURSP1 and pURSP2 (*22*) were used to transform nmt81-*oma2*^+^ cells. Approximately 106 400 individual transformants were further selected on thiamine-containing medium. After a second round of selection, library plasmids were recovered and retransformed into strain nmt81-*oma2*^+^. Genes of interest were subcloned into the vector pUR19 (*22*), and individually validated as suppressors of nmt81-*oma2*^+^. Among the identified suppressors were the homeobox protein SPAC32A11.03c/Phx1p, which is essential for long-term survival and meiotic sporulation (*37*), the GDP-mannose-1-phosphate guanylyltransferase SPCC1906.01/Mpg1p required for proper septum structure and cell cycle progression (*38*), and SPAC637.06/Gmh5p, a so far uncharacterized predicted α-1,2-galactosyltransferase (*15*, *39*) (Fig. 2a). Phx1p (SPAC32A11.03c) is a transcription factor essential for long-term survival and stress adaptation in *S. pombe* (*37*). Its multicopy suppression of O-mannosyltransferase mutants may result from enhanced stress tolerance and metabolic flexibility, indirectly supporting cell viability under glycosylation stress. Overexpression of SPCC1906.01/Mpg1p, which encodes a GDP-mannose-1-phosphate guanyltransferase essential for GDP-mannose biosynthesis also suppressed the lethal phenotype of mutant oma2Δ (Fig. 2b). This effect is most likely due to increased availability of this donor substrate for various glycosylation steps.

In contrast to *mpg1*^+^, *gmh5*^+^ overexpression does not suppress the lethality of oma2Δ (Fig. 2b). The different outcomes of the complementation tests (compare Fig. 2a and Fig. 2b) can be explained by residual expression of *oma2*^+^ in the thiamine-repressible strain due to promoter leakiness, while in the oma2Δ mutant, Oma2p activity is completely lost after 5’-FOA treatment. Consequently, suppressor *gmh5*^+^ can only complement the thiamine-repressed strain, but not the full deletion of *oma2*^+^. Furthermore, hypersensitivity of the viable oma1Δ and oma4Δ mutants to the drug caffeine (*18*) is significantly reduced when *gmh5*^+^ is overexpressed, although not to full extent. Apparent IC_50_ (half maximal inhibitory concentration) values change from 4.30 to 6.81 mM in oma1Δ, and from 5.00 to 7.17 mM in oma4Δ mutants expressing *gmh5*^+^ on pREP3adh, but are still below the IC_50_ value of 8.55 mM in WT cells (Fig. 2c). Based on these findings, we consider *gmh5*^+^ to be a moderate suppressor of O-mannosylation deficiency. In the following study we are focusing on Gmh5p.

### Phylogenetic and structural analysis of Gmh5p

SPAC637.06/Gmh5p is a member of the glycosyltransferase family 34 (GT34), including the *S. pombe* glycosyltransferases Gma12p and Gmh1p to Gmh6p (*40*). These enzymes show structural features typical for type-II Golgi glycosyltransferases with a single transmembrane domain flanked by a short N-terminal cytoplasmic and a C-terminal catalytic domain and a typical DxD motif for divalent metal ion binding (*41*). Gmh1p, Gmh2p, Gmh3p, Gmh6p and Gma12p are confirmed α-1,2-galactosyltransferases acting in the extension of N-linked outer chain and O-mannosyl glycans (*15*, *16*). Gmh5p was initially predicted to act as α-1,2-galactosyltransferase. However, *in vivo* galactosylation of N- and O-linked glycans was unaffected in a *S. pombe gmh5*^+^ deletion mutant (*15*); thus, the enzymatic activity of Gmh5p remains elusive.

**Fig. 3:**
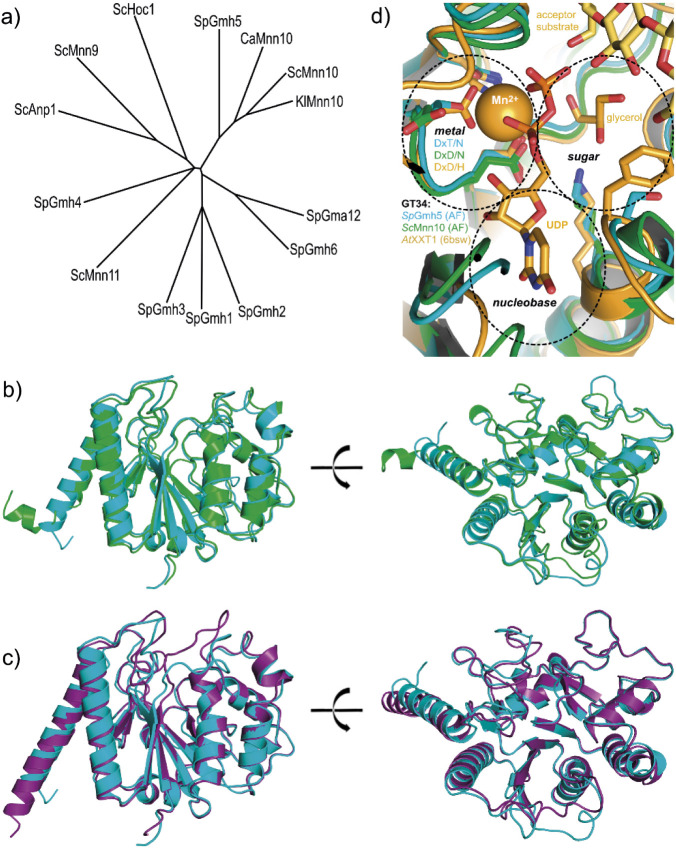
Phylogenetic analysis and structural alignments of Gmh5p with GT34 family glycosyltransferases: a) phylogeny of galactosyl- and mannosyltransferases. Phylogenetic analysis of protein sequences of various galactosyl- and mannosyltransferases from different yeast strains revealed the highest homology of Gmh5p to α-1,6-mannosyltransferases. Protein sequences from *S. pombe* (Sp), *S. cerevisiae* (Sc), *Candida albicans* (Ca), and *Kluyveromyces lactis* (Kl) were used, b) superimposition of Gmh5p (cyan) and α-1,6-mannosyltransferase Mnn10p from *S. cerevisiae* (green), c) superimposition of Gmh5p (cyan) and α-1,2-galactosyltransferase Gma12p from *S. pombe* (deep purple). The Gmh5p, Mnn10p and Gma12p structures are AlphaFold predictions with a very high (pLDDT>90) score, which is highly reliable for a defined fold. N-terminal and C-terminal tails are truncated for clarity, and d) active site superimpositions within the GT34 family for Gmh5p (cyan), Mnn10p (green), and AtXXT1 (orange, PDB ID: 6bsw). Functions are assigned according to AtXXT1 (dashed ovals): Sugar-binding site indicated by a bound glycerol molecule, metal binding site by the DxD/H motif and nucleobase pocket by the uridine moiety. The active site view corresponds to the right panels in b) and c)

The GT34 family includes the mannosyltransferases Mnn10p and Mnn11p from *S. cerevisiae* (*42*, *43*). Phylogenetic analysis of protein sequences of various galactosyl- and mannosyltransferases of this family from different yeast species revealed the highest homology of Gmh5p to Mnn10p α-1,6-mannosyltransferases (Fig. 3a), with a sequence identity of 38 % to Mnn10p from *S. cerevisiae*, compared to only 29 % identity to the α-1,2-galactosyltransferase Gma12p from *S. pombe*.

Like other GT34 family members, Gmh5p is predicted to be a retaining glycosyltransferase with a GT-A fold, including a Rossmann-like domain (*40*, *44*). Gmh5p features a DxD-like motif for divalent metal ion binding, although the second aspartate is replaced by threonine (DxT), a variation also observed in other glycosyltransferases (*44*). Currently the only GT34 enzyme with an experimentally determined 3D structure is the xyloglucan-xylosyltransferase 1 (XXT1; PDB ID: 6bsu) from *Arabidopsis thaliana* (*45*), but no experimental structures are available for fungal GT34 enzymes. However, AlphaFold (*29*) predictions provide highly reliable models of the core domains for Gmh5p, Mnn10p, and the confirmed α-1,2-galactosyltransferase Gma12p (*46*). Structural superimposition of Gmh5p and Mnn10p yields a root mean square deviation (RMSD) of 1.2 Å over 265 Cα atoms, indicating a very close structural similarity (Fig. 3b). In contrast, the RMSD between Gmh5p and Gma12p is 1.5 Å over 219 Cα atoms (Fig. 3c), reflecting greater divergence. A detailed comparison of the active sites within the GT34 family shows that in contrast to XXT1, Gmh5p and Mnn10p are nearly identical in their active site architecture, especially in the nucleotide-binding site (Fig. 3d). All enzymes share the GT-A fold and the metal-binding motif, suggesting similar mechanisms for donor substrate binding. However, there is a key difference in nucleotide specificity of the enzymes: while XXT1 from *Arabidopsis* uses UDP-xylose (*47*), Mnn10p uses GDP-mannose (*9*) as a donor substrate. Overall, the nucleotide-binding site of Gmh5p is highly similar to Mnn10p, suggesting that Gmh5p uses GDP-mannose rather than UDP-galactose as donor substrate and is thus more likely to act as a mannosyl- than a galactosyltransferase.

### Gmh5^+^ acts as mannosyltransferase

**Fig. 4:**
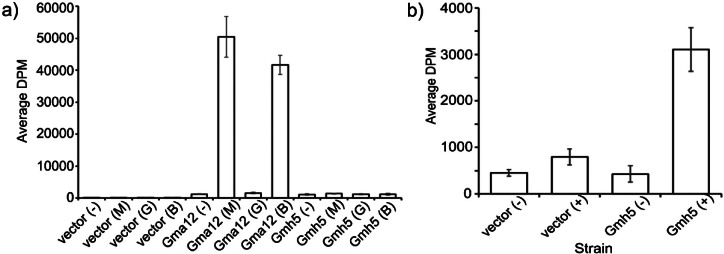
Galactosyl- and mannosyltransferase activity of Gmh5p. Gmh5p-HA and the proven galactosyltransferase Gma1p-HA, respectively, were expressed in and enriched from *S. cerevisiae* wild-type (WT) cells (Fig. S2a and Fig. S2b): a) galactosyltransfer from UDP-[^3^H]galactose to α-methyl-d-mannoside (M), α-methyl-d-galactoside (G), and α-1,6-mannobiose (B) was determined. *S. cerevisiae* cells transformed with the empty vector served as a control. Enriched membranes served as enzyme source. In strains overexpressing Gma12p, transferase activity to α-methyl-d-mannoside (Gma12 (M)) and α-1,6-mannobiose (Gma12 (B)) was significantly increased compared to the other conditions tested (p<0.0001), and b) mannosyltransferase activity of purified Gmh5p-HA was determined with GDP-[3H]mannose as donor in the presence (+) or absence (-) of α-methyl-d-mannoside as an acceptor. Immunopurified Gmh5p-HA served as enzyme source. Experiments were performed three times using three replicates. Average transferase activities from all experiments are shown. Gmh5p transferase activity (Gmh5 (+)) was significantly increased compared to the other conditions tested (p<0.0001). DPM=disintegrations per minute.

**Fig. S2:**
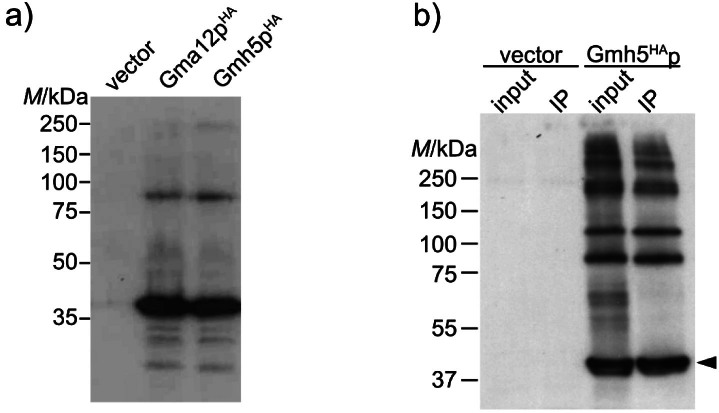
Expression of *S. pombe* Gmh5p and Gma12p in *Saccharomyces cerevisiae*: a) *S. pombe* Gmh5p-HA (pCM11), Gma12p-HA (pGma12HA) and an empty vector control were expressed in *S. cerevisiae* wild-type (WT) cells. Microsomal membranes equivalent to *A*_600 nm_(cell solution)=1 were separated on 10 % SDS-polyacrylamide gels, and b) Gmh5p-HA was solubilized from microsomal membranes using 1 % digitonine and immunopurified using anti-HA monoclonal antibodies coupled to sepharose beads using standard procedures. Aliquots of the input and the immunoprecipitate were separated on 10 % SDS-polyacrylamide gels. HA-tagged proteins were detected by Western blot decorated with monoclonal mouse anti‐HA (16B12; Abcam, Cambridge, UK) at 1:8000 dilution. IP=immunoprecipitate, HA=hemagglutinin

Since baker's yeast does not possess intrinsic galactosyltransfease activity, we further explored the enzymatic activity of Gmh5p in *S. cerevisiae*. For this purpose, Gmh5p and Gma12p, C-terminally tagged with the hemagglutinin (HA) epitope, were recombinantly expressed under the control of the *adh1* promoter in *S. cerevisiae* WT strain BY4741 (Fig. S2a). Microsomal membranes were used as an enzyme source to measure galactosyl transfer from UDP-[3H]galactose to the putative acceptor substrates α-methyl-d-galactoside, α-methyl-d-mannoside and α-1,6-mannobiose. As shown in Fig. 4a, galactosyltransfer by Gma12p-HA to α-methyl-d-mannoside and to α-1,6-mannobiose could be detected, confirming previous results. However, Gmh5p-HA showed no activity towards the tested acceptor substrates. Thus, the results further support that Gmh5p does not act as α-1,2-galactosyltransferase.

To test for mannosyltransferase activity, Gmh5p-HA was solubilized from microsomal membranes using 1 % digitonine and immunopurified using anti-HA monoclonal antibodies attached to sepharose beads. Immunopurified Gmh5p-HA catalyzed the transfer of α-[3H]mannose from GDP-[3H]mannose to α-methyl-d-mannoside, which serves as a standard model substrate in such assays (Fig. 4b and Fig. S2b). These data further support mannosyltransferase activity Gmh5p.

These experimental results validate our structural predictions and clarify the previous ambiguity regarding the enzymatic specificity of Gmh5p. The activity of Gmh5p corresponds to its closer sequence and structural similarity to Mnn10p from *S. cerevisiae*, an α-1,6-mannosyltransferase. This provides conclusive evidence that Gmh5p functions as a mannosyl- and not as a galactosyltransferase.

### Gmh5^+^ represents an orthologue of S. cerevisiae mnn10

**Fig. 5:**
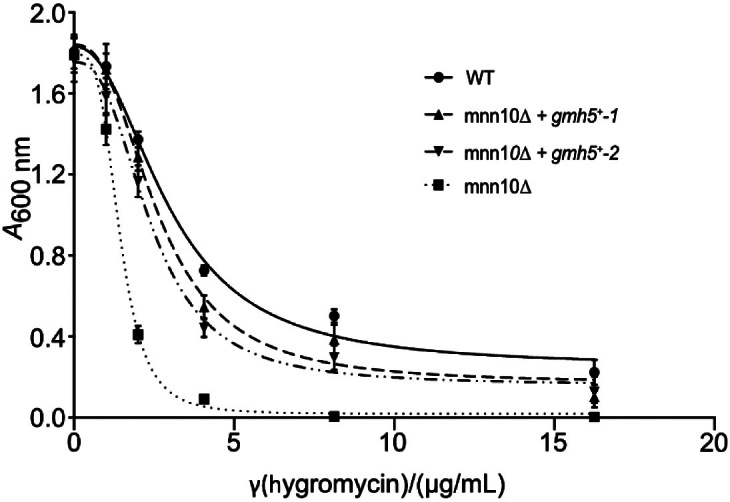
*Complementation of S. cerevisiae* mutant mnn10Δ with *S. pombe gmh5*^+^. *S. cerevisiae* mnn10Δ mutant transformed with a *gmh5*^+^ expression plasmid or the empty vector backbone, as well as the isogenic wild-type (WT) strain transformed with the empty vector, were treated with the indicated concentrations of hygromycin. Absorption at *A*_600 nm_ was measured after a growth period of 24 h. Strains mnn10Δ + *gmh5*^+^-1 and mnn10Δ + *gmh5*^+^-2 represent two independent transformants. The experiment was performed twice using three technical replicates. Results were used for linear regression analysis and the IC_50_ was determined. Differences in the IC_50_ values were confirmed to be significant by statistical analysis (p<0.05)

Having defined Gmh5p as a mannosyltransferase, we examined whether Gmh5p is a functional homologue of *S. cerevisiae* Mnn10p. The Mnn10p protein is a major catalytic subunit of the Golgi-localized M-Pol II complex (Anp1p, Hoc1p Mnn9p to Mnn11p), responsible for the elongation of the α-1,6-mannan backbone of N-linked carbohydrates in baker's yeast (*42*). In *S. cerevisiae*, *mnn10* mutants have a severely negative impact on N-linked glycosylation (*48*) and show strong sensitivity towards the aminoglycoside hygromycin B (*43*). This drug sensitivity was exploited to determine the functional conservation between *S. cerevisiae* Mnn10p and *S. pombe* Gmh5p. To that end, Gmh5p was expressed in *S. cerevisiae* mnn10Δ mutant cells. As shown in Fig. 5, hygromycin tolerance in mnn10Δ strains expressing *gmh5*^+^ nearly reached WT levels (IC_50_: mnn10Δ, 1.34 µg/mL; mnn10Δ + *gmh5*^+^, 2.515 µg/mL (two individual transformants are shown in Fig. 5), WT, 3.00 µg/mL), qualifying Gmh5p as an Mnn10p orthologue.

This functional conservation between Gmh5p and Mnn10p is particularly interesting given their taxonomic distance. *S. pombe* and *S. cerevisiae* diverged approximately 400-600 million years ago and have distinct cell wall compositions (*49*, *50*). The ability of Gmh5p to complement the mnn10Δ phenotype supports the conclusion that core aspects of N-glycan biosynthesis pathways remain conserved despite other evolutionary changes in cell wall structure and composition (*12*, *51*). Interestingly, in *S. cerevisiae* pmtΔ mutants, the Mnn10p orthologue Mnn11p (21 % sequence identity) was shown to be destabilized in pmtΔ mutants, most likely due to reduced O-mannosylation (*52*). A similar mechanism may apply in *S. pombe*, where impaired O-mannosylation in omaΔ mutants could affect the stability of Gmh5p and thereby explain its role as a multicopy suppressor of omaΔ mutants. Exploring this hypothesis in *S. pombe* will be an interesting avenue for future research.

### Gmh5p participates in N-linked glycan outer chain elongation

**Fig. 6:**
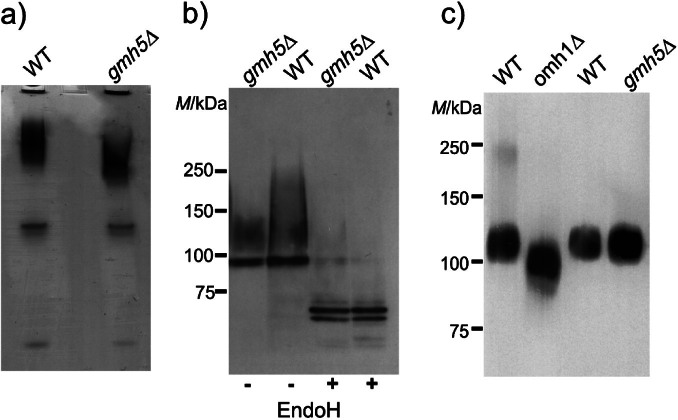
Analysis of glycosylated proteins in mutant gmh5Δ: a) acidic phosphatase: the enzyme was induced in wild-type (WT) and gmh5Δ, and analyzed on polyacrylamide native gels as described in Materials and Methods, b) invertase: a recombinant version of *S. cerevisiae* invertase was expressed in mutant gmh5Δ and the isogenic WT strain. Protein extracts were treated with endoglycosidase H (Endo H) to remove N-linked glycans and analyzed by Western blot using invertase-specific antibodies (see Materials and Methods for details), and c) chitinase: *S. cerevisiae* chitinase was heterologously expressed in *S. pombe* omh1Δ and gmh5Δ mutants and the isogenic WT strain. Chitinase was extracted from medium supernatants using crab shell chitin and analyzed by Western blot using chitinase-specific antibodies (detailed in Materials and Methods). Mutant omh1Δ, lacking a α-1,2-mannosyltransferase which plays a major role in extending O-linked mannoses, served as a control

In order to investigate changes in protein glycosylation in more detail, we created an *S. pombe gmh5*^+^ deletion strain (detailed in Materials and Methods). The known N-glycosylated *S. pombe* protein, acidic phosphatase (*53*) was induced in WT and gmh5Δ mutant strains. Cell lysates were analyzed by native polyacrylamide gel-electrophoresis followed by activity staining. Acid phosphatase extracted from gmh5Δ cells has increased electrophoretic mobility compared to WT cells (Fig. 6a), suggesting decreased N-glycosylation, which agrees with Gmh5p acting as an *S. cerevisiae* Mnn10p orthologue in the elongation of N-linked core glycans. In addition, the highly N-glycosylated and O-mannosylated *S. cerevisiae* enzymes invertase (*54*) and chitinase (*24*), respectively, were recombinantly expressed in *S. pombe* mutant gmh5Δ. Enriched protein fractions were isolated and proteins analyzed as described in Materials and Methods. Both enzymes were detected with protein-specific polyclonal antibodies. Like in *S. cerevisiae*, in *S. pombe* WT cells, invertase migrates as a broad band ranging from 100 to 250 kDa, which shifts to the mass of the unmodified protein (~60 kDa) after N-glycans were removed with Endo H (Fig. 6b). In mutant gmh5Δ, high molecular mass protein species >150 kDa are strongly reduced, which further corroborates the role of Gmh5p in the extension of outer chain of N-glycans. In contrast, the molecular mass of exclusively O-mannosylated chitinase is not changed in mutant gmh5Δ compared to WT (Fig. 6c). The observation that O-mannosylation of chitinase remains unaffected in gmh5Δ mutants while N-glycosylation of acid phosphatase and invertase is significantly impacted highlights the specificity of Gmh5p in the biosynthesis of N-linked glycans.

In *S. pombe*, N-linked outer chains can be elongated by the action of an M-Pol II-like complex composed of Mnn9p and Anp1p, which sequentially elongates the mannose chains (*12*). This complex is mirroring the function of M-Pol I (Anp1p and Van1p) and M-Pol II (including Anp1p, Mnn10p, Mnn11p and Hoc1p) in *S. cerevisiae* (*8*, *9*, *42*) but may differ in its assembly. Our data now strongly support that Gmh5p is an orthologue of *S. cerevisiae* Mnn10p and contributes to M-Pol II-like complex activity in *S. pombe* by further extending the mannose backbone. This is evident in Fig. 6a and Fig. 6b, where Western blot shows that although N-glycosylation of acid phosphatase and invertase is still present, the glycan chains are substantially shorter, as indicated by the reduced molecular mass of the glycoproteins. Whether these effects observed in mutant gmh5Δ can be complemented by heterologous expression of *S. cerevisiae* Mnn10p remains to be determined.

**Fig. 7:**
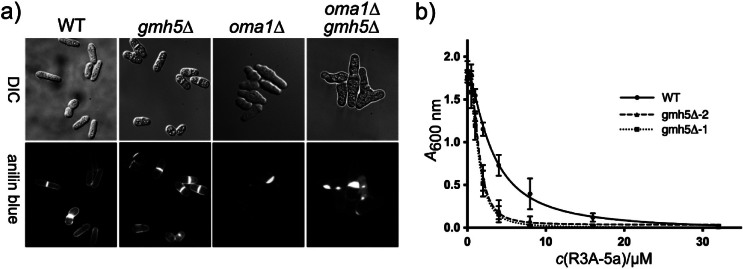
Phenotypic characterization of *S. pombe* gmh5Δ mutants: a) wild-type (WT) and the indicated mutant strains were grown to logarithmic phase on EMM medium (for details see Materials and Methods). Mutant oma1Δgmh5Δ shows defects in cell separation leading to abnormally shaped elongated or branched cells. Aniline blue staining is used to visualize β-glucans. Differential interference contrast (DIC) and fluorescence microscopic images are shown; magnification 630-fold, and b) treatment of *S. pombe* strains with the O-mannosylation inhibitor R3A-5a. WT and two independent mutant clones (gmh5Δ-1 and gmh5Δ-2; represented as dotted and dashed lines, respectively), were treated with the indicated concentrations of R3A-5a. *A*_600 nm_ was determined after a growth period of 24 h. The experiment was performed twice using three technical replicates. Results were used for linear regression analysis and the IC_50_ was determined. Differences in the IC_50_ values were confirmed to be significant by statistical analysis (p<0.05)

In *S. cerevisiae*, impairment of the M-Pol II complex, as in anp1Δ mutants, leads to synthetic growth defects under conditions of compromised O-mannosylation (*55*). In line with its function as an orthologue of *S. cerevisiae* Mnn10p, targeted disruption of *gmh5*^+^ in oma1Δ cells resulted in increased phenotypic severity of aberrant cell and cell wall morphologies (Fig. 7a). Furthermore, gmh5Δ mutants were highly susceptible to the protein O-mannosyltransferase inhibitor R3A-5a (*23*) (IC_50_: WT, 3.08 µM; gmh5Δ, 1.45 µM (two individual mutant clones are shown in Fig. 7b).

The exacerbated phenotypic severity observed when *gmh5*^+^ is disrupted in oma1Δ cells shows an important interplay between O- and N-linked glycosylation pathways in maintaining cell wall integrity. Similarly, the hypersensitivity of gmh5Δ mutants to the inhibitor R3A-5a suggests a compensatory relationship between these pathways. Our previous work in *S. cerevisiae* already showed a compensatory response between the two types of glycosylation, specifically in the formation of high mannose outer chains. Transcription of several mannosyltransferase genes involved in the biosynthesis of N-linked high mannose carbohydrate chains was induced upon treatment with O-mannosylation inhibitors and in pmtΔ mutants (*56*). Similarly, inhibition of N-glycosylation by tunicamycin leads to increased transcription of *PMT*s and *KTR1* involved in O-glycosylation (*57*), further suggesting a regulatory relationship between these pathways in yeast cell wall biogenesis.

## CONCLUSIONS

Our data strongly indicate that Gmh5p is involved in the outer chain elongation of N-linked glycans, almost certainly representing the *Saccharomyces cerevisiae* Mnn10p orthologue. Our identification of Gmh5p as a multicopy suppressor of decreased O-mannosylation highlights the complex interplay between different glycosylation pathways in maintaining cell wall integrity. The characterization of Gmh5p as a mannosyltransferase rather than the previously predicted galactosyltransferase contributes to our understanding of glycan biosynthesis in *Schizosaccharomyces pombe* and reveals evolutionarily conserved aspects of protein glycosylation between distantly related yeast species. These findings provide important insights into the critical role of protein glycosylation in cell wall integrity and the function of a previously uncharacterized glycosyltransferase.

## SUPPLEMENTARY MATERIALS

All supplementary materials are available at: www.ftb.com.hr.

## Appendix

**Table S1:** Yeast strains used in this study

Strain	Genotype	Reference
*S. pombe*		
FY527	h^–^, *ade6-M216, his3-*Δ*1*, *leu1-32*, *ura4-*Δ*18*	Forsburg lab
SBY90	Isogenic to FY527 except *oma1*::*hisG*	(*18*)
SBY88	Isogenic to FY527 except *oma4*::*hisG*	(*18*)
TWY14	*ade6-M210*, *his3-*Δ*1*, *leu1-32*, *ura4-*Δ*18*, *oma2::his3^+^*, transformed with pTW51-1	(*18*)
TWY16	Isogenic to FY527 except nmt81-*oma2*^+^	(*18*)
CMY1	Isogenic to FY527 except *gmh5*::*kanMX*	This study
CMY2	Isogenic to SBY90 except *gmh5*::*kanMX*	This study
omh1Δ	h^+^, *ade6-M210*, *ura4-*Δ*18*, *leu1-32*, *omh1*::*kanMX4*	(*14*)
*S. cerevisiae*		
BY4741	MATa *his3*Δ*1*, *leu2*Δ*0*, *met15*Δ*0*, *ura3*Δ*0*	Euroscarf, Oberursel, Germany
mnn10Δ	Isogenic to BY4741 except *mnn10*::*kanMX*	Euroscarf, Oberursel, Germany
